# Sucrose affects the developmental transition of rhizomes in *Oryza longistaminata*

**DOI:** 10.1007/s10265-018-1033-x

**Published:** 2018-05-08

**Authors:** Kanako Bessho-Uehara, Jovano Erris Nugroho, Hirono Kondo, Rosalyn B. Angeles-Shim, Motoyuki Ashikari

**Affiliations:** 1 Bioscience and Biotechnology Center, Nagoya University, Furo-cho, Chikusa, Nagoya, Aichi 464-8601 Japan; 2 Department of Plant and Soil Science, Texas Tech University, Lubbock, TX 79409 USA

**Keywords:** Rhizome, *Oryza longistaminata*, Sucrose, Developmental transition, Gravitropism

## Abstract

*Oryza longistaminata*, the African wild rice, can propagate vegetatively through rhizomes. Rhizomes elongate horizontally underground as sink organs, however, they undergo a developmental transition that shifts their growth to the surface of the ground to become aerial stems. This particular stage is essential for the establishment of new ramets. While several determinants such as abiotic stimuli and plant hormones have been reported as key factors effecting developmental transition in aerial stem, the cause of this phenomenon in rhizome remains elusive. This study shows that depletion of nutrients, particularly sucrose, is the key stimulus that induces the developmental transition in rhizomes, as indicated by the gradient of sugars from the base to the tip of the rhizome. Sugar treatments revealed that sucrose specifically represses the developmental transition from rhizome to aerial stem by inhibiting the expression of sugar metabolism and hormone synthesis genes at the bending point. Sucrose depletion affected several factors contributing to the developmental transition of rhizome including signal transduction, transcriptional regulation and plant hormone balance.

**Electronic supplementary material:**

The online version of this article (10.1007/s10265-018-1033-x) contains supplementary material, which is available to authorized users.

## Introduction

**Fig. 1:**
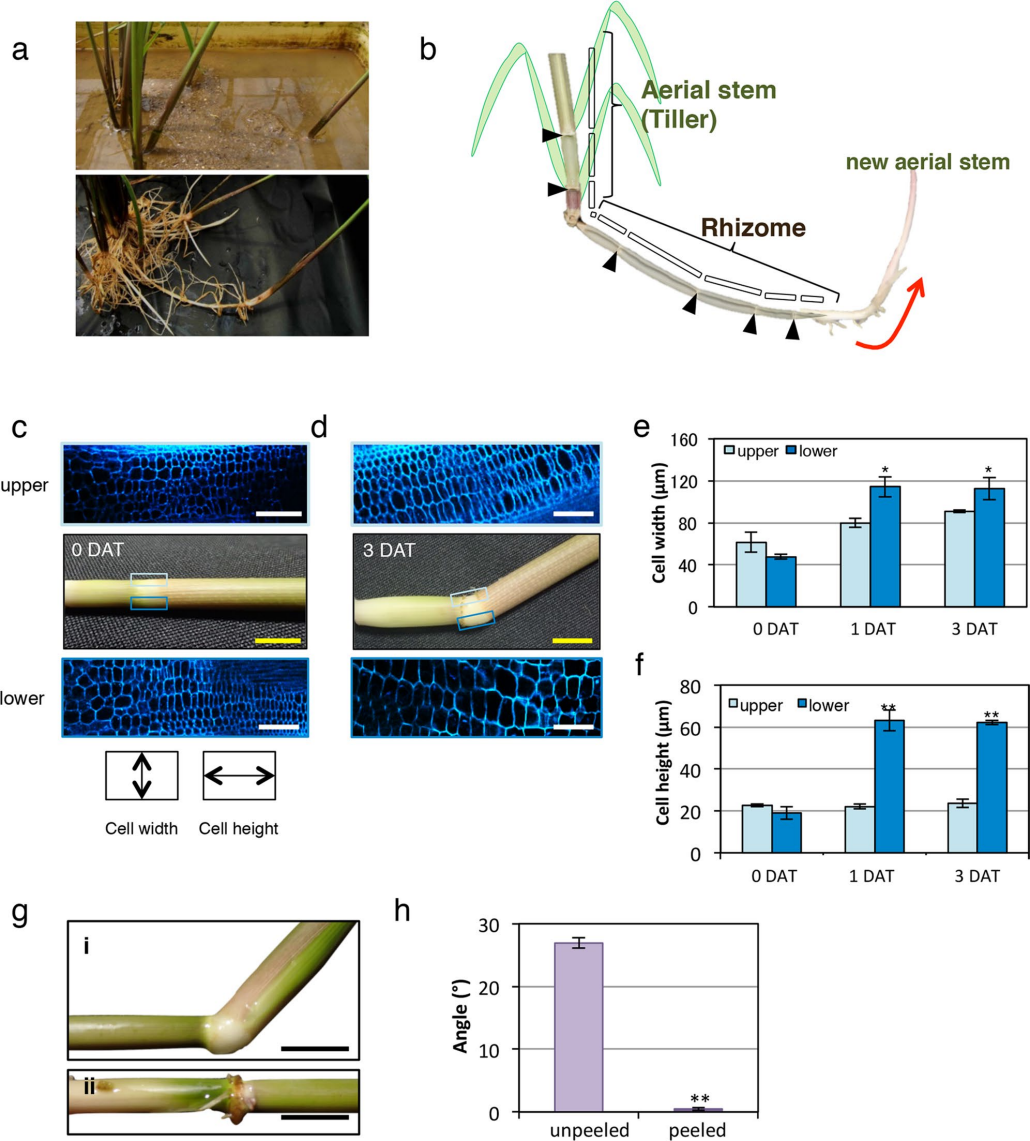
Morphological changes at the bending point of the aerial stem. **a** Upper picture observed from soil surface showing a new tiller as ramet growing far from the parental shoot of *O. longistaminata*. Uncovering the soil, lower picture showing the separated tiller is connected to the parental shoot through rhizomes. **b** Image of aerial stem and rhizome of *O. longistaminata*. Aerial stem and rhizome have similar phytomer structure. Each black box represents one unit of phytomer separated by nodes (indicated by black triangle). Red arrow indicates the direction of developmental transition from rhizome to new aerial stem. **c**–**f** Gravi-response of aerial stem. Comparison of cell sizes on the upper and lower segments of pulvini (yellow scale bars = 1 cm, white scale bars = 100 µm.) Pulvinus structure and longitudinal section of aerial stems before (**c**) and 3 days after orienting the aerial stems to lie onto its side (**d**). Gray and blue rectangles indicate the upper and the lower segment. The graph shows the width and height of cells from 60 cells of 3 plants (**e, f**). **g** The node around the region with (i) and without (ii) pulvinus 3 days after orienting the aerial stems to lie onto its side. **h** The graph shows bending angle from five plants. *DAT* days after treatment, Error bars indicate ± SD. Significant differences were detected by student t test, **p ≤ 0.01, *p ≤ 0.05

Plants ensure the continuation of its own kind by either sexual or asexual reproduction. Asexual reproduction could be via vegetative propagation wherein a part of the parental plant gives rise to a new plant that is genetically identical to that of the parent. An example is the potato which forms tubers at the tip of stolons as sink organs. Buds on these tubers can give rise to new individuals. Bamboo, on the other hand, propagates through rhizomes which elongate underground. Rhizomes transport water and nutrients, and are interconnected with each aerial stem. Some of the axillary buds on the rhizome develop into new aerial stems. Although bamboo sprouts appear on the surface of the ground in spring with the changes in temperature and day length, almost nothing is known about the molecular mechanism that allows the developmental transition from rhizome to aerial stem. *Oryza longistaminata* A.Chev. & Roehrich, also known as red rice or long-stamen rice, is an endemic wild rice species in Africa that propagates mainly via rhizome formation. It belongs to the species complex with AA genome where *O. sativa*, a model monocot plant, also belongs (Khush 1997). *O. longistaminata* grows to about 2 m in height and propagate all year round and propagates through the rhizomes (Fig. 1a), whereas bamboos grow around 10 m high and develop new sprouts that appear only for a limited time. This makes *O. longistaminata* suitable for the study of developmental transition in rhizome.

Morphological observations revealed that although rhizomes grow underground, its their anatomical features closely resemble aerial stems. The presence of a phytomer structure of nodes interspaced with internodes that have hollow structures in both rhizomes and aerial stems further support this similarity (Fig. 1b). Although rhizomes, as well as tillers, can develop from axillary buds, the morphology of a rhizome bud is completely different from that of a tiller bud. Rhizome bud in *O. longistaminata* is onion-like-shaped and develops in an oblique direction (Yoshida et al. 2016). Time course observation revealed that the rhizome bud penetrates the surrounding leaf sheath and then the internodes elongate under the ground (Supplementary material 1). After elongation, the rhizomes acquire negative gravitropism and start moving aboveground to develop as a new individual aerial stem (Supplementary material 2, Fig. 1b). Each stage of rhizome development seems to be regulated by many factors. Especially during the final stage of upward growth, the rhizome undergoes several changes to attain negative gravitropism along with the developmental transition from rhizome to aerial stem.

Many factors related to negative gravitropism and developmental transition in aerial stem have already been reported (Clore 2013; Collings et al. 1998; Wong and Colasanti 2007). For instance, the phytohormone auxin have been shown to accumulate asymmetrically in barley aerial stem, thereby upregulating the expression of gibberellin (GA) synthesis genes and inducing negative gravitropism (Ross and Wolbang 2008; Wolbang et al. 2004). Abiotic stimuli such as anoxic environments affect stem gravitropism of aerial stem in maize, barley and rice (Azuma et al. 2013). As for the rhizome, light exposure, as well as auxin and GA has been reported to influence the shooting of rhizome in the rhizomatous plants, *Elymus repens* (McDowell and Gang 2013). Yet, the molecular mechanisms driving the induction of negative gravitropism and developmental transition of rhizomes remain largely unknown. Recently, sucrose has also been reported to affect the developmental transition of *O. longistaminata* rhizome (Fan et al. 2017). Fan et al. (2017) suggested that sucrose might suppress the negative gravitropism of rhizome and keep rhizomatous growth by inhibiting the transition to aerial stem. However, it has not been verified how sucrose affect the developmental transition of *O. longistaminata* rhizome and whether other sugars play a role in this transition. In the first place, it is unknown how the rhizome of *O. longistaminata* bends and when the developmental transition would occur. In this study, we investigated the timing of developmental transition of *O. longistaminata* rhizome and where the negative gravitropic growth occurred in rhizome by morphological observation. Further we determined the specific effects of sucrose on the developmental transition of *O. longistaminata* rhizome and how it connects with expression patterns of the genes related to gravitropism.

## Materials and methods

### Plant materials and growth conditions

The plant used for the experiments is *O. longistaminata* Acc. IRGC110404 from the International Rice Research Institute (IRRI), Los Baños, Laguna, Philippines. The plant materials were grown in the greenhouse at Nagoya University, Aichi under 16 h of light/8 h of darkness, and an average temperature of 28.4 °C.

### Time course observation

Concentrated powder of Otsuka house 1, 2 and 5 fertilizers diluted 1,000 times (OAT Agrio Co.) was used for the hydroponic culture media. *O. longistaminata* plants that were vegetatively maintained in the greenhouse were transferred in an acrylic box filled with liquid media. The box was covered with a corrugated cardboard and the whole set-up was surrounded with a black curtain so that light would not penetrate the underground part. A green LED was used to illuminate the underground part when taking photos used to make a movie on rhizome development. The whole setup was placed in a controlled environment chamber (NKsystem LP-2P) set to 16 h of light/8 h of darkness, average humidity of 65.7%, and an average temperature of 30.4 °C. Brinno TLC200 was used to take Supplementary material 1, and Canon EOS 40D was used to take Supplementary material 2.

### Aerial stem gravity stimulation

The main, vertically straight, aerial stems were collected, washed and cut 6 cm above the first node with a razor blade while the roots were kept intact. The stem segments were then oriented horizontally with the basal portion placed in the middle of two plastic plates fastened together using rubber bands. The plates were then placed in a styrofoam box filled with 1–2 cm of distilled water solution and kept inside a dark incubator set at 28 °C. Recorded pictures (taken with Olympus TG-4) at 0, 1 and 3 days were compared to get the angle of upward bending of the aerial shoots. The main, vertically straight, aerial stems which have at least one internode were used for the peeling experiment. The leaf sheath attached to the lowest node was peeled and carried out the gravity stimulation that was performed same as above. The angle was measured 3 days after the treatment. We used five plants for the morphological observation of aerial stem.

### Microscopic observation and cell size measurement

**Fig. 2:**
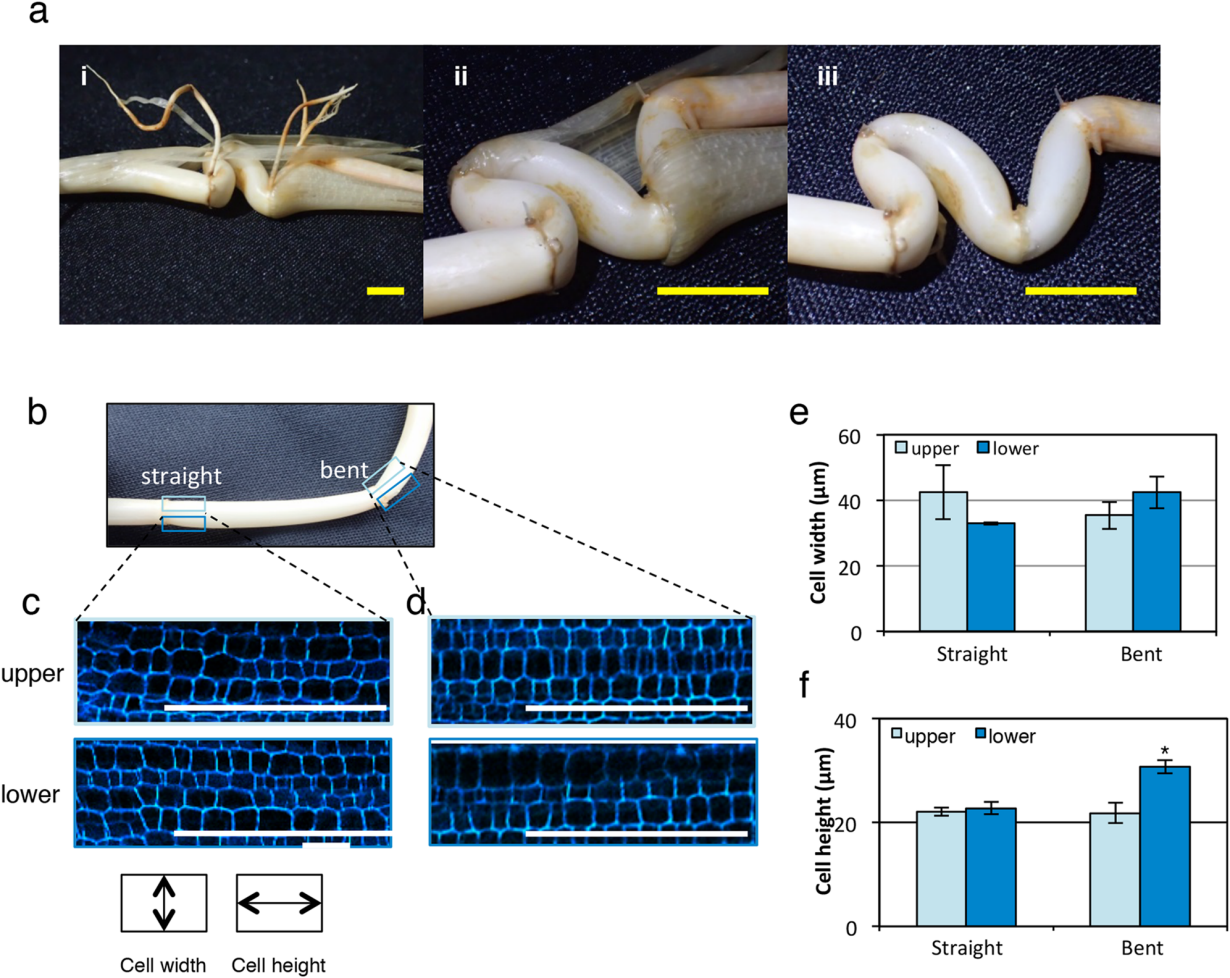
Morphological changes at the bending point of the rhizome. **a** Twisted rhizome underground with scale leaves (i), magnified picture (ii), and without scale leaves (iii). Yellow scale bar = 2 cm. **b** The appearance of normal bending rhizome, each rectangle colored in gray and blue indicate the upper and lower segments that were sectioned in **c, d. c**–**f** Comparison of cell sizes on the upper and lower segments of the rhizome. White scale bars = 100 µm. Rhizome structure and longitudinal section of its straight part (**c**) and bending part (**d**). The graph shows the width and height of cells from 60 cells of 3 samples (**e, f**). Error bars indicate ± SD. Significant difference was detected by student t test, *p ≤ 0.05

The pulvinus of aerial stem or the internode of rhizomes were cut to approximately 1 cm length. The samples were then soaked in a fixative (10% paraformaldehyde (PFA)/60 mM 4-(2-hydroxyethyl)-1-piperazineethanesulfonic acid (HEPES)/0.5 M sucrose), centrifuged at 4 °C at 5,000*G* for 2 min and stored at 4 °C. After 2 h, the fixative was removed and replaced with 1 × PBS buffer solution. Samples were centrifuged again at 4 °C at 5,000*G* for 2 min. This buffer exchange step was repeated three times. The fixed samples were used immediately for microscopic observations or stored at 4 °C. The segments were sliced 120 µm thick with a micro-slicer (DSK Microslicer^™^ DTK-3000W) set at 70% frequency. The specimens were placed on micro slide glass S2441 and layered with a micro cover glass (Matsunami) and observed under a fluorescence stereoscopic microscope. Cell size measurement was performed using the ImageJ software (Version 1.44). We used aerial stem after gravistimulation in Fig. 1, naturally bent rhizome in Fig. 2, and rhizome that was bent by physical compression from the growing apparatus in Fig. S2.

### Time required for the developmental transition from rhizome to aerial stem

Rhizomes with five internodes which are still growing underground were used. After measuring the rhizome length, the samples were placed back into the soil and allowed to grow until the rhizome tip appears in the ground surface. The number of days needed for the developmental transition from rhizome to aerial stem to occur was recorded.

### Starch content quantification

Primary rhizomes with five internodes were cleaned and rid of their scale leaves. Five separate sections of the internodes were soaked in commercial iodine gargle solution (including KI-I_2_) diluted 40 times with shaking for 30 min. The segments were washed with distilled water. The ends of the segments were then sliced (± 1 mm) and photographed. The picture was analyzed and quantified by reversed plot profiling using the ImageJ software (Version 1.44).

### Internal sugar measurement

Primary rhizomes with five internodes that are still growing underground were cleaned and rid of scale leaves. Starting from the basal part, the 1st, 3rd and 5th internodes were separated and placed in liquid N_2_, then crushed to fine powder with a mortar. Ground samples (0.15 g) were transferred into an Eppendorf tube and mixed in 1 ml miliQ water. After vortexing, the samples were then centrifuged at 4 °C at 5,000*G* for 4 min. Only the supernatant was used for sugar measurement. Sucrose, d-glucose and d-fructose concentrations were determined using the UV method with reagents and protocol supplied by the Enzymatic BioAnalysis of Boehringer Manheim/R-Biopharm.

### External treatments of sugar, GA and UNI to rhizome

Primary rhizomes with five internodes that have not been exposed to light were cleaned with distilled water, their basal parts removed and placed in 15 mL falcon tubes, which have been opened on both ends. The tubes were filled with control solution ($\frac{1}{2}$ MS liquid media) or sugar solution ($\frac{1}{2}$ MS liquid media + 0.1% (w/v) sucrose/d-glucose/d-fructose/maltose) for exogenous sugar treatments. For the GA or GA biosynthesis inhibitor [uniconazole (UNI); inhibitor of ent-Kaurene oxidase] treatments, 10^− 5^M GA_3_ and 10^− 6^M UNI were used with or without 0.1% (w/v) sucrose. Both ends of the tube were sealed with parafilm. The samples were placed in a humid chamber under dark condition at 28 °C. The solutions were renewed every 24 h from the hole of the falcon tube. All treatments were replicated in at least three independent biological experiments. The sugar treatment was carried out for 8 days, and the GA and UNI treatments were performed for 4 days. Pictures were taken using an Olympus TG-4, iSO 200, 2.4 magnification under green light. The angle of curvature growth was calculated using the ImageJ software (Version 1.44).

### Carbon isotope fractionation

Samples were prepared using the same protocol for external sugar treatment with several alterations. In the case of the negative control, 50% nutrient mix + 0.1% (w/v) sucrose was used. For C-13 sucrose treatment [UL-13Cglc] + [1-13Cfru] 0.1% (w/v) mixture was applied (Omicron Biochemicals, Inc.) d-glucose isotopic label 1,2-13C2—enrichment 99% and d-fructose isotopic label 1-13C—enrichment 99% were utilized for C-13 glucose and C-13 fructose, respectively (Cambridge Isotope Laboratories, Inc.) After 3 days of treatment, regions 1 cm above and below the youngest nodes were collected, subjected to cryodesiccation for 24 h and burnt for gas chromatography–mass spectrometry analysis by SI Science Co., Ltd. δ^13^C is an isotopic signature used to measure the ratio of stable isotopes ^13^C:^12^C expressed as parts per thousand or per mil (‰). The formula for δ^13^C in per mil is:

$${{\varvec{\updelta}}}13{\text{C}}=\left( {\frac{{\left( {\frac{{13{\text{C}}}}{{12{\text{C}}}}} \right){\text{sample}}}}{{\left( {\frac{{13{\text{C}}}}{{12{\text{C}}}}} \right){\text{standard}}}} - 1} \right) \times \permille,$$

where the standard is an established reference material.

### RNA extraction and quantitative RT-PCR

The bending part (near the 5th node from the base) of the rhizome was used for qRT-PCR analysis of bending-related genes after an 8-h treatment with or without 0.1% sucrose. Total RNA was extracted from all samples by RNeasy Plant Mini Kit (QIAGEN). First-strand cDNA synthesis was performed using the Omniscript RT Kit (QIAGEN). StepOneTM Real-Time PCR system (Applied Biosystems) was used to analyze the relative expression levels of the target genes. Relative expression levels of the target genes were normalized to the levels of endogenous ubiquitin transcripts (*OsUBI*). Each set of experiments was repeated three times, and the Comparative CT method (ΔΔCT Method) was used to calculate the relative expression levels of the target genes. The sequence of genes used for qRT-PCR was referred from the genome sequence of *O. longistaminata* read by our group (unpublished). Primer sequence used in this experiment is in Table S1.

## Results

### Differential cell size in bending region occur during negative gravitropism in aerial stem and rhizome of *O. longistaminata*

To investigate the cause of the negative gravitropic response which occurs prior to/or along with the developmental transition in rhizome, we first compared the gravitropism between aerial stems and rhizomes. Because of the morphological similarities between these two organs (Fig. 1b), it is thought that rhizomes would show a gravitropic response similar to that of the aerial stem. We initially observed the response of *O. longistaminata* aerial stems to gravity change by orienting *O. longistaminata* aerial stems to lie onto its side (Fig. S1a). After 1- and 3-day treatment, the aerial stems curved upwards at a 15° and 20° angle at the pulvinus region, respectively (Fig. S1b, c). The pulvinus is a part of the basal leaf sheath in aerial stems that is essential for gravity-induced bending response in monocot aerial stems (Dayanandan and Kaufman 1984). Longitudinal sections of the pulvinus region of excised aerial stems before and after gravistimulation were observed (Fig. 1c, d) to compare the cell width and height on the upper (gray rectangle) and lower (blue rectagle) segment of the pulvinus. Microscopic observations showed no difference in the size of the cells in the upper and the lower segment of the pulvinus before gravistimulation (0 DAT, Fig. 1e, f). After 1- and 3-days of gravistimulation, however, cells in both the upper and lower segment of the pulvinus were wider than before gravistimulation (1DAT and 3DAT, Fig. 1e) and cells in the lower segment of the pulvinus were on average, three times higher (about 60 µm) compared to cells from the upper segment of the pulvinus (about 20 µm) (1DAT and 3DAT, Fig. 1f). Difference in cell sizes were maximum after 1 day of gravity stimulation.

To understand the pulvinus effect on aerial stem bending, we compared the differences in the gravitropic response of *O. longistaminata* aerial stem with peeled and unpeeled pulvinus. Aerial stem with unpeeled pulvinus showed regular negative gravitropic response while that with a peeled pulvinus did not show any gravitropic response (Fig. 1g, h). These data indicate that the pulvinus is essential for the stem’s gravity response, and that the aerial stem of *O. longistaminata* shares the same mechanism for negative gravitropism with those of other monocot species (Cosgrove 1997; Song et al. 2014).

Interestingly, the scale leaves surrounding the rhizome internodes do not have pulvini. Observing a rhizome which twisted underground but did not go upward, we anticipated that the rhizome can bend without pulvini (Fig. 2a). Same as aerial stem, a test whether the rhizome bending occurred or not depending on the existence of scale leaves was carried out. The rhizomes with peeled scale leaves could bend as easily as unpeeled one (Fig. S2a). This indicates that the scale leaves without pulvini are not crucial for rhizome bending as opposed to the importance of pulvinus in aerial stems for gravity-induced bending. Instead, probably the internode itself could bend in the rhizome. The part of internodes located just above the node exhibit remarkable bending ability under natural conditions. Microscopic investigations were similarly carried out to measure the cell height and width of longitudinal sections of straight rhizome segments (taken close to the base of rhizome) and bent segments (taken from relatively near the tip of rhizome) (Fig. 2b). Cell size measurements showed no differences in the width and height of cells at the upper and lower segments of the straight rhizome region (straight, Fig. 2c, e, f). However, in the bent rhizome region, the height of the cells at the lower segment was significantly higher by 10 µm (bent, Fig. 2d–f). In addition, the twisted rhizome showed differences in cell height measured inside and outside of the internode (Fig. S2b). These results suggest that the rhizomes have achieved overall flexion ability by pushing through cell expansion at the lower side of internode at bending point to move upward via increases in cell height.

### The determinants of developmental transition in rhizome are related with the number of internodes

**Fig. 3:**
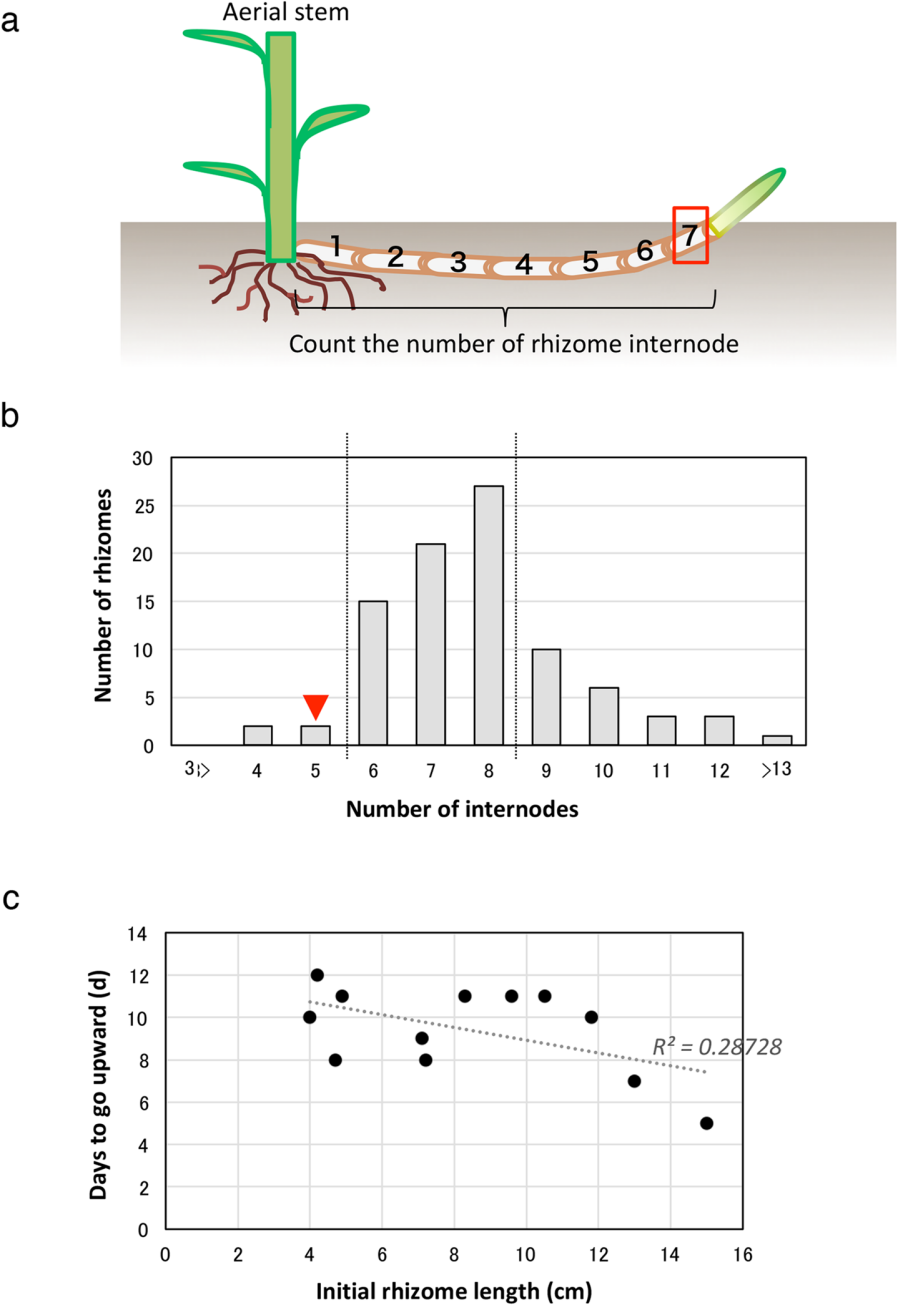
Relationship between the morphological feature of rhizome and rhizome bending. **a** Illustration on how the number of internodes of already bent rhizome was counted. Red rectangle indicates the bending point. **b** Graph showing the elongated internode number in rhizome from base to bending point of rhizome. Bars within the dotted lines show 71% of rhizome has elongated 6th to 8th internodes underground. Red triangle indicates the 5-internode rhizome used for the physiological experiments (Figs. 4, 5, 6). **c** Graph showing the correlation between rhizome initial length and the number of days required for the rhizome to aerial stem aboveground

Bending occurs at different parts of aerial stems and rhizomes. Bending of the aerial stem is basically a gravity-dependent phenomenon that occurs with the change of gravity direction, but that of the rhizome seems to depend on time or developmental stage (Supplementary material 2). To determine what causes bending and developmental transition, we counted the number of internodes in the rhizome which are already coming up aboveground (Fig. 3a). From 90 rhizomes investigated, 71% exhibited bending from the 6th to the 8th internodes before it curved and pushed above ground (Fig. 3b). This result indicates that the signal that activates the negative gravitropism and developmental transition of rhizomes occurs around the 6th to the 8th internodes.

**Fig. 4:**
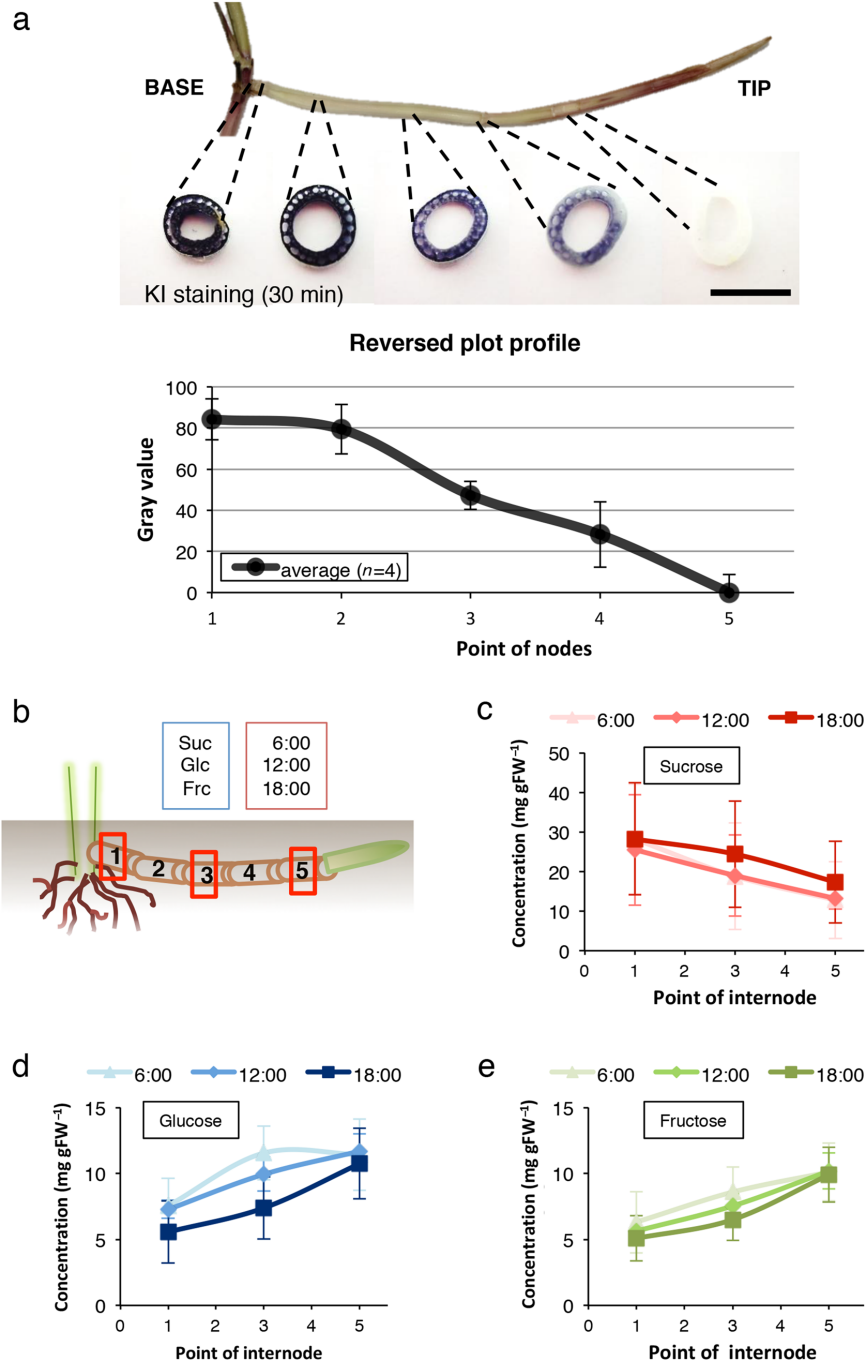
Carbohydrates distribution in *O. longistaminata* rhizome. **a** Transverse sections of rhizomes after 30 min of staining with KI solution. *Scale bar* = 5 mm. Reversed plot profile of staining intensity was analyzed using the ImageJ software (Ver1.44). Error bars indicate ± SEM for four biological replicates. **b** Illustration of rhizome sampling condition. Sampling points were at the 1st, 3rd and 5th internode indicated by red rectangle. *Suc* sucrose, *Glc* glucose, *Frc* fructose. Sugar concentration in the rhizome; sucrose (**c**), glucose (**d**), fructose (**e**). Error bars indicate ± SD for five biological replicates

**Fig. 5:**
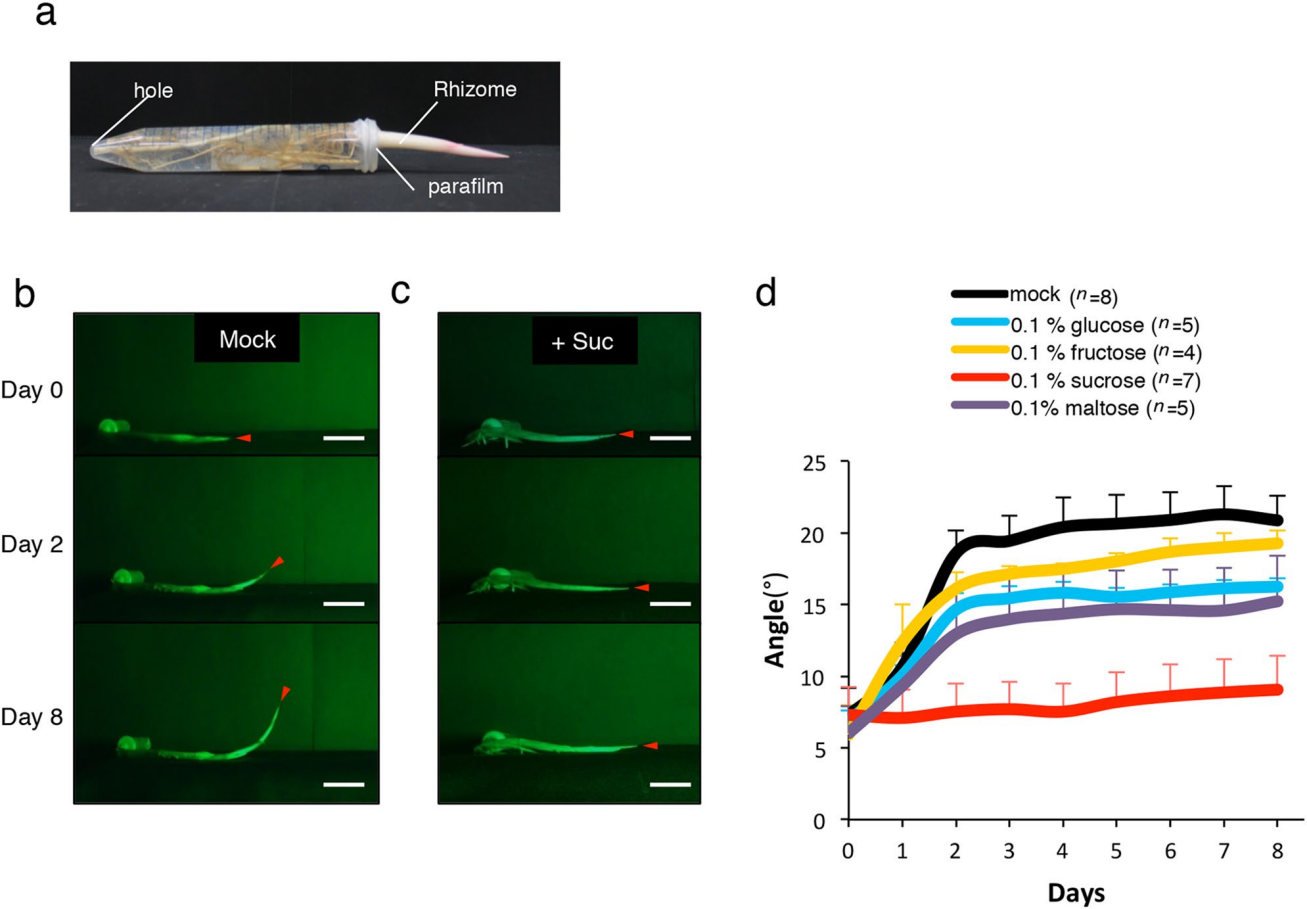
Effect of sugars on the bending response of excised rhizomes. **a** Image of setup for sugar treatments. **b, c** Physiological observations of rhizomes during external sugar treatments. *Scale bars* = 3 cm, red triangle pointing at the tip of the rhizome. Mock (**b**) and with sucrose (**c**). **d** Change in the angle of upward growing rhizomes during sugar treatments. Error bars indicate ± SEM

**Fig. 6:**
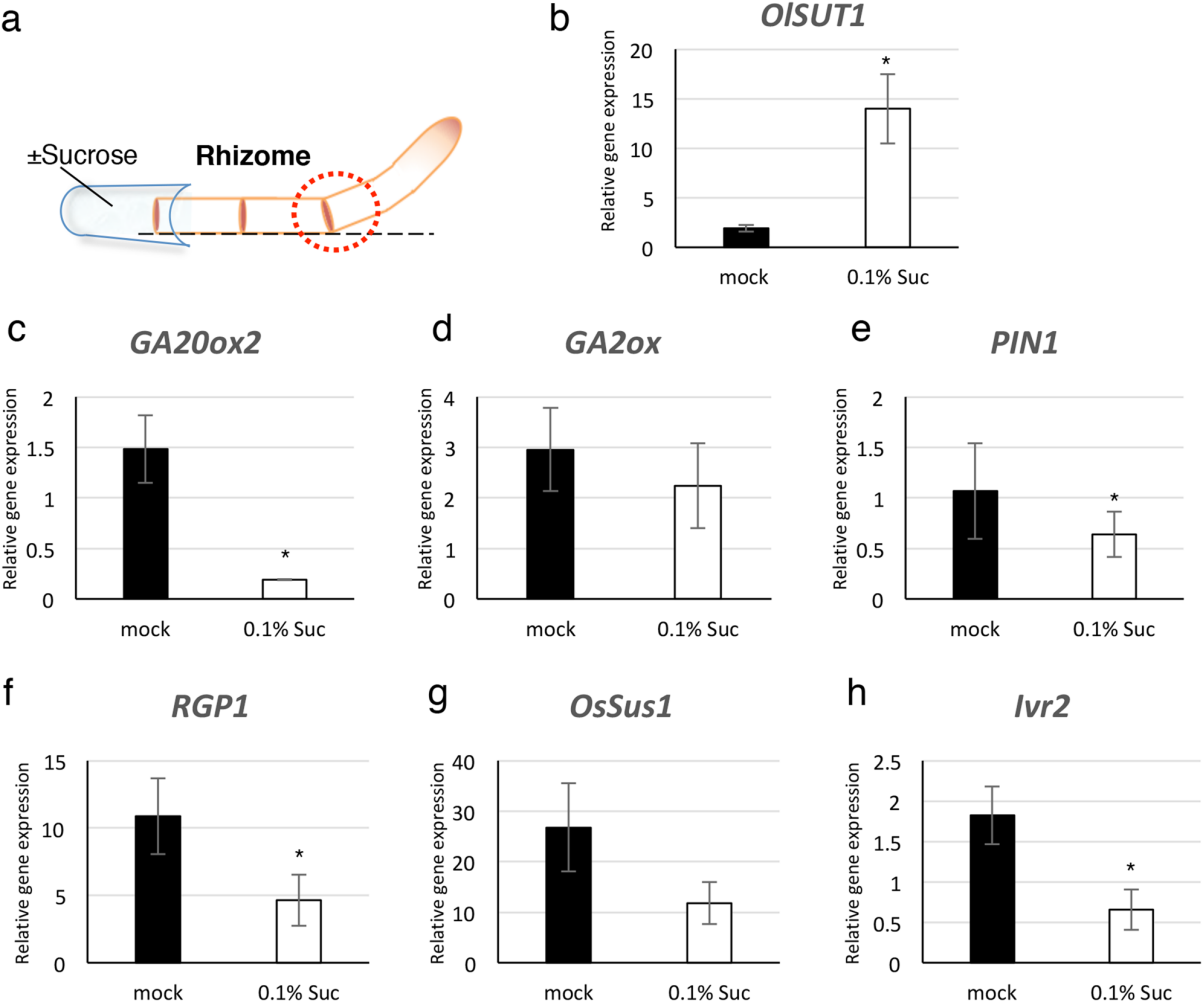
Changes in the relative expression levels of genes after sucrose treatment in the bending part of rhizome. **a** The sampling point of rhizome with or without sucrose treatment. **b**–**g** Expression levels of developmental transition-related and gravitropic response-related genes, *OsSUT1* (**b**), *GA20ox2* (**c**), *GA2ox* (**d**), *OsPIN1* (**e**), *RGP1* (**f**), *OsSUS1* (**g**) and *Ivr2* (**h**) in *O. longistaminata* rhizome. Black and white bar indicates without or with sucrose, respectively. *Suc* sucrose. Values are represented relative to the expression level of *OsUBI* transcript. Error bars indicate ± SD for five biological replicates. Significant differences were detected by student t test, *p ≤ 0.05

Subsequently, to verify whether the length of the rhizome and the developmental transition into aerial stem are linked or not, we measured the time it took for the tips of differently sized rhizomes to appear in the ground surface. The results showed no correlation between the length of the rhizome and the time it took for the rhizomes to change into aerial stems (*R* ^2^ = 0.28728, Fig. 3c). This result indicated that the number of internodes and not the total length of rhizome is important when rhizomes undergo the developmental transition into aerial stems. It seems that some cues affect the developmental transition in rhizome along with the number of internodes and rhizomes can time its upward movement. We used primary rhizomes having five internodes that were still growing under the soil to further investigate the factors causing the developmental transition in rhizome.

### The distribution of carbohydrates in rhizome changes throughout rhizome development

Among many aspects that contribute to the developmental transition in rhizome, we focused on examining the effects of nutrients, particularly sugars. Sugars are known as mobile signals for gravitropism both in the roots and in the shoots (Sack 1997; Song et al. 1988). Moreover, plants carry out photosynthesis predominantly in their leaves to produce mainly sugars, which are then transported throughout the plant body including the rhizomes as a sink organ (Bel and Hess 2008). Sucrose and carbon metabolism are important for plant development and are regulated depending on the cell type and developmental stage. Sucrose metabolism in particular, is affected by both internal and external environmental signals that can alter development and stress acclimation response in plants (Figueroa and Lunn 2016; Koch 2004). Based on these reports, we looked at the sugar distribution in rhizomes during the developmental transition from rhizome to aerial stem.

To test this hypothesis, we first observed the starch accumulation pattern in rhizomes because starch is the major storage form of carbohydrates. The internodes of the rhizome located just above the 1st to the 5th nodes were stained with I_2_-KI (KI staining) to measure starch concentration using the ImageJ software. The basal part of the rhizome recorded the highest colored value of 80, whereas the tip recorded the lowest colored value of zero (Fig. 4a). This result indicated a decreasing concentration of starch from the base to the tip of rhizome. The concentrations of three major transportable saccharides namely sucrose, glucose and fructose, were measured at three regions of the rhizome (1st, 3rd and 5th internodes). Sampling was performed at different time points (at 6:00, 12:00 and 18:00) in 1 day to investigate the diurnal patterns of sugar accumulation (Fig. 4b). Sucrose concentration decreased from the base to the tip of the rhizome similar to the concentration patterns observed for starch (Fig. 4c), whereas glucose and fructose concentration showed a slightly increasing trend (Fig. 4d, e). Gradients in sugar concentration were observed regardless of the time of day. In the case of rhizomes that already emerged aboveground and presumably received photosynthates from two sources (two ramets), sucrose accumulated in the middle section of rhizomes, whereas glucose and fructose were barely detected (Fig. S3a, b). As nitrogen is also important to determine the path of bud development as a shoot or rhizome (McIntyre 2001), we could not detect the nitrogen concentration in *O. longistaminata* rhizome this time (data not shown). These results suggest that sugar accumulation in rhizome changes with the developmental growth of the rhizome itself and that sugars may act as signaling or regulating molecules.

### The negative gravitropism and developmental transition of rhizomes is repressed by sucrose

To further understand the effect of sugars on the developmental transition of rhizomes, we performed external sugar treatments. To exclude the other factors that might affect the developmental transition of rhizomes, aerial stems were removed, leaving only the rhizomes and parts of the root system (Fig. 5a). Since starch was not soluble, it was not used this time. This experiment was performed under dark conditions, with minimum use of green light when taking pictures to eliminate the influence of light. Our investigation showed that mock treatment ($\frac{1}{2}$ MS liquid media) of rhizomes resulted in steep curvature growth of up to 20° angle after 2 days of treatment (Fig. 5b, d). In contrast, rhizome treatment with 0.1% sucrose completely repressed the upward growth of the rhizome even after 8 days of treatment (Fig. 5c, d). Since maltose is often used as a component of osmotic hypertonic solution, it was used as a control. Application of glucose, fructose and maltose did not suppress the upward bending of rhizomes but resulted in a slightly lower angle of 13°–17° (Fig. 5d). Although sugars other than sucrose also have small inhibitory effects on the bending and developmental transition of the rhizomes, it was shown that sucrose exerts these effects most notably. We concluded that sucrose has the strongest potential to repress the upward bending of rhizomes.

To confirm whether sucrose, glucose and fructose are absorbed by rhizomes, we performed Carbon-13 (13C) isotopic labeling for external sugar treatments. Based on the reference standard established for 13C using the Cretaceous marine fossil, *Belemnitella americana*, most natural material would show a negative δ^13^C value (Coleman and Fry 1991). We propose that a δ^13^C value higher than 10 ‰ compared to the negative control would indicate a biological significance in the absorption. Sucrose, glucose and fructose treatments showed δ^13^C value higher than 10 ‰ (Table 1), implying that they were indeed taken up by rhizomes. Fructose had the highest δ^13^C value average (102.93 ‰), followed by glucose at 55.11 ‰ and sucrose at 35.04 ‰, although a wide variation was observed in the values obtained for glucose. Based on these results, we conclude that sucrose, and not the other sugars, specifically represses the developmental transition and negative gravitropism of rhizome although all the three sugars were absorbed into rhizomes.

**Table 1:** 13C labeling sugars absorbance into excised rhizome

No.	Sample name	^13^C	^13^C/^12^C		δ^13^C (‰)
		(Atom%)	Std	Sample	
			0.011237		
1	Sucrose negative control_1	1.0736		0.01085	
2	Sucrose negative control_2	1.0735		0.01085	
3	Sucrose negative control_3	1.0746		0.01086	
4	Sucrose-13_1	1.109		0.01121	33.35
5	Sucrose-13_2	1.1101		0.01123	34.38
6	Sucrose-13_3	1.1108		0.01123	35.04
7	Fructose-13_1	1.1771		0.01191	97.55
8	Fructose-13_2	1.1751		0.01188	95.01
9	Fructose-13_3	1.1828		0.01197	102.93
10	Glucose-13_1	1.1321		0.01145	55.11
11	Glucose-13_2	1.1197		0.01132	43.43
12	Glucose-13_3	1.0897		0.01102	15.17

### Sucrose represses several genes related to the developmental transition

Several plant hormones have been reported to be related to the plant developmental transition. For example, a reduction in the endogenous levels of bioactive gibberellin (GA) in a maize mutant delays the developmental transition from juvenile vegetative to adult vegetative stage (Evans and Poethig 1995). GA is known to be as important as auxin in promoting negative gravitropism in the aerial stem of many plant species (Li et al. 2007; Rakusová et al. 2011; Rood et al. 1987; Ross and Wolbang 2008). However, the interaction between GA related genes and sucrose remains unclear. To investigate the alteration in the transcript level of GA synthesis, we measured the expression level of GA synthase *GA20ox*, and GA metabolizing gene *GA2ox* at the bending point of rhizome after sucrose or non-sucrose treatments (Fig. 6a). *OsSUT1* (sucrose transporter) was used as positive control and it was upregulated by sucrose treatment (Fig. 6b). *GA20ox2* has been reported to be strongly expressed in *O. longistaminata* rhizome (Yoshida et al. 2016), so we chose this allele for the analysis. Since polar and lateral transport of auxins are also important for the asymmetrical auxin distribution in the bending part of the stem and root of many plants in response to gravity (Philosoph-Hadas et al. 2005), the expression of the auxin carrier *OsPIN-LIKE 1* (*OsPIN1*) was also examined. The results showed that *GA20ox2* and *OsPIN1* showed significantly lower expression with sucrose treatments compared to the control, whereas changes in the level of *GA2ox* expression were not observed (Fig. 6c–e). These results indicated that both GA and auxin were kept at low levels in the bending part of the rhizome by sucrose.

Several other genes have been reported to contribute to developmental transition and negative gravitropism. Previous studies showed that *OsRGP1* and *OsSus1* showed asymmetric expression pattern in *O. sativa* aerial stem with gravity stimulation (Hu et al. 2009). Both genes decompose sucrose and the moiety is used for cell wall synthesis. The new cell walls are needed for the high cell division and cytoskeleton rearrangement (Gunning and Sammut 1990). Upregulation of these genes suggests the activation of cell proliferation. Significantly lower expression of *OsRGP1* was observed at the bending point of the rhizome whereas the expression of *OsSus1* tends to be repressed with sucrose treatment (Fig. 6f, g). The expression level of *Invertase2* (*Ivr2*), an enzyme that catalyzes the hydrolysis of sucrose, was downregulated at the bending part of the rhizome with sucrose treatment (Fig. 6h). In the pulvini of oat shoots, both expression and activity of invertase were higher in the lower side of the gravitropic responding organs compared to the upper side (Wu et al. 1993). These results indicate that sucrose represses GA synthesis, auxin transport and cell wall synthesis directly or indirectly, then induce the repression of developmental transition from rhizome to aerial stem.

### GA promotes the negative gravitropism of rhizome in *O. longistaminata*

**Fig. 7:**
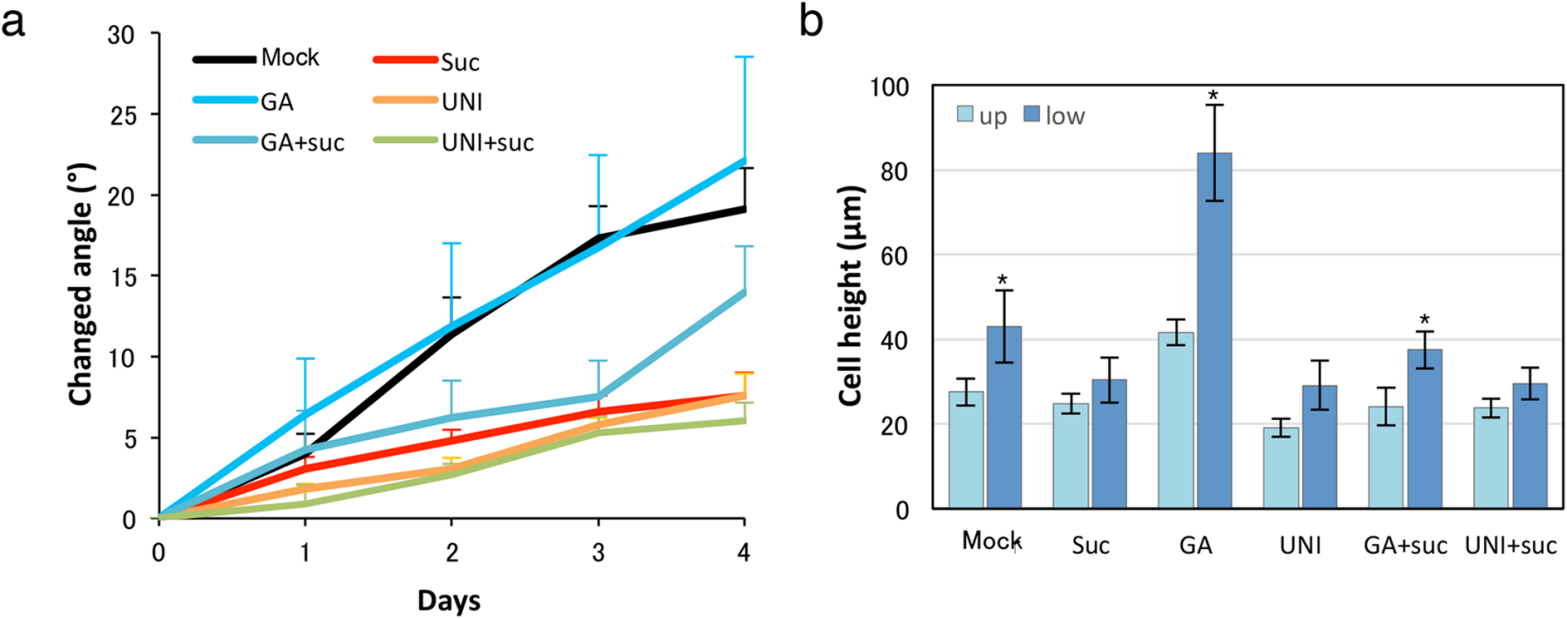
GA overcomes the sucrose effect on negative gravitropism of rhizome by promoting cell elongation. **a** Change in the angle of upward growing rhizomes during GA and UNI treatments with or without sucrose. Error bars indicate ± SEM. **b** Cell height at upper and lower part of the bending position of rhizome. Twenty cells of three plants were measured. Significant difference was detected by student t test, *p ≤ 0.05. *Suc* sucrose, *GA* gibberellin, *UNI* uniconazole

To understand how GA relates to the bending of rhizome, GA and its inhibitor, uniconazole (UNI), were applied to rhizomes with or without sucrose. GA treatment enhanced the curvature growth on the first day and prolonged the change of the rhizome angle (Fig. 7a). On the other hand, UNI repressed the upward growth of the rhizome the same as the sucrose treatment. Further, the rhizomes treated with GA + sucrose showed a curvature change depend on the treatment duration while rhizomes treated with UNI + sucrose maintained horizontal growth. These results suggest that GA overcame the sucrose effect on rhizome bending while UNI enhanced it. Anatomical observations revealed that GA clearly affected cell height (Fig. 7b). Although GA promotes cell elongation twice as much as control, it did not have a significant effect on the bending angle between both treatments. It was probably because the cell height ratio in the upper and lower part of the rhizome treated with GA (1.91) was almost the same as the control (1.68). The cell height in the lower part of the rhizome treated with GA + sucrose showed a significant difference compared with the cell height in the upper part of the rhizome. This result suggests that GA compensates rhizome bending by cell elongation even in the presence of sucrose. UNI treatment showed an antagonistic effect compared with GA treatment on cell elongation. However, there was no significant difference for UNI treatment, intending to enhance the effects of sucrose in repressing cell elongation in the lower part of the rhizomes.

## Discussion

Both rhizomes and aerial stems are histologically classified into stems, but their morphological features and developmental mechanisms are different. According to Yoshida et al. (2016), the rhizomes have leaves consisting solely of the leaf sheath, called scale leaves attached to each phytomer. Here we show that the pulvini, an essential organ for aerial stem bending, is absent on the scale leaves of rhizome, and that the rhizome bends at the internode itself (Fig. 2a). This observation suggests that the mechanisms involved in gravitropism in rhizomes and aerial stems are different. The cell size at the lower side of the internode is higher than that of the upper side at the bending point of a rhizome. This suggests that cell expansion at the lower side of the internode at the bending point allows rhizomes to move aboveground.

Among the many factors that are proposed to regulate negative gravitropism along with the developmental transition of rhizome, we focused on the effect of nutrients, particularly sugars. Many studies have shown that sugars can act as signaling molecules and/or global regulators of gene expression, for instance, acting like hormones to regulate developmental growth and floral transition (Coneva et al. 2012; Figueroa and Lunn 2016:Koch 2004). Both sucrose abundance and depletion could modulate growth at the whole plant level and change tissue- or cell-specific transcription patterns (Cookson et al. 2016; Eveland and Jackson 2012). Because sucrose concentrations in the rhizomes decreased from the base to the tip, it is possible that depletion of nutrients from the parental plant activates the developmental transition in the rhizome. Although starch, also a product of photosynthesis, shows a similar gradient in a rhizome from the base to the tip, its decomposition to sucrose and other sugars might be required for the growth of the rhizome and/or production of new cell walls. Similar results on the effects of sucrose on the developmental transition of *O. longistaminata* rhizome has recently been reported (Fan et al. 2017). Based on these knowledge, we speculated that sucrose might have an influence on the gravitropic response of rhizome during its developmental transition towards becoming an aerial stem. Our results indicate that decreasing sucrose concentration might be one factor controlling this developmental transition and the negative gravitropism in rhizome (Fig. 4c). Reduction in sucrose concentration in the rhizome was observed regardless of sampling time, although the concentration of glucose and fructose were slightly higher in the morning. This is possibly because both glucose and fructose are exported during the day or accumulate at night by decomposing sucrose (Lemoine et al. 2013). External application of sucrose also repressed the bending of rhizomes (Fig. 5c). Interestingly, while the introduction of glucose, fructose and maltose mildly repressed bending, sucrose distinctly inhibited the upward growth of the rhizome (Fig. 5d). Is rhizome bending repressed by the osmotic pressure created by the sugar treatment? Monosaccharides have higher osmolarity than disaccharides, nevertheless, sucrose completely repressed rhizome bending, indicating that the repression of rhizome bending due to sucrose application was not merely due to different osmotic effects.

Rhizome bending was clearest on the second day (Fig. 5d), indicating that molecular changes at the bent region of the rhizome occurred in less than 48 h after sugar application. Previous reports showed that many transcript changes occur within 30 min after carbon depletion (Cookson et al. 2016). According to this, there is time lag between gene expression and visible phenotype change. The expression levels of genes related to hormone synthesis/transport and sugar metabolism were assessed to determine the effects of sucrose on the developmental transition of rhizomes after 8-h sugar treatment. Auxin is a well-known plant hormone that regulates negative gravitropism by accumulating asymmetrically at the bending point of aerial stems (Li et al. 2007; Philosoph-Hadas et al. 2005; Wolbang et al. 2004). Auxins also promote biosynthesis of the bioactive GA by upregulating GA synthesis gene expression in many plant species (O’Neill and Ross 2002; Wolbang et al. 2004). These indicate the presence of auxin–GA interaction during gravity response. GA is reported to overcome the inhibition of rhizome bending by sucrose in the rhizomatous bermudagrass (Montaldi 1969) because it activates the invertase transcription to promote cell wall production. Thus, we investigated the expression levels of genes related to bending and developmental transition after sucrose treatment.

*OsPIN1, GA20ox2* and *Ivr2* expressions at the bending point of the rhizome were lower with sucrose treatment compared to the control but no change in *GA2ox*, GA-deactivated gene, was observed. These results suggest that sucrose represses auxin transport and GA synthesis, eventually leading to the downregulation of invertase expression. Further investigation on phytohormone distribution, mainly auxin and GA, at the rhizome bending point has to be considered and more experiments that would verify the relationship between auxin, GA and rhizome bending (i.e. simultaneous application of auxin and GA with sucrose, or auxin and GA inhibitors treatment) are necessary in future works. *SUS1* has been reported to be involved in regulating osmotic pressure and turgor during rapid cell elongation (Pfeiffer and Kutschera 1995). This gene was upregulated at the bending point of monocot pulvini to promote the decomposition of sucrose and the production of UDP-glucose, a material for cell wall synthesis (Long et al. 2002; Persia et al. 2008). *RGP1*, a UDP-arabinopyranose mutase, has been implicated in the interconversion of UDP-Arap, whereas UDP-Araf has been associated with the cell wall intensification of pollen tube (Drakakaki et al. 2006). In this study, *OsRGP1* was significantly repressed and *OsSus1* was mildly repressed at the bending part of the rhizome by sucrose treatment. This result suggests that auxin transport was repressed and these genes were subsequently downregulated. These genes are related to the construction of new cell walls indicating new cell production. Sucrose causes downregulation of *OsSUS1* and *OsRGP1* and it suggests that the cell division is repressed at bending point of rhizome by sucrose treatment. We showed that cells were longer at the lower part of the bent rhizome (Fig. 2), although there is no indication of direct evidence of activation of cell division in this part of the rhizome. EdU staining experiments are necessary to verify whether cell division is activated or not during bending.

In contrast to the well-known roles of auxin in gravitropic response, the role of GA synthesis in rhizome developmental transition is uncertain. Previous studies showed the antagonistic effect of GA on rhizome development, promotion or inhibition. Kumar and Wareing (1972) reported that GA can promote horizontal elongation of potato stolon underground, while Montaldi (1969) indicated that GA can inhibit horizontal rhizome growth in bermudagrass. These reports suggested that the effect of GA on rhizome development was valuable depending on plant species. Treatment of rhizomes with GA and its inhibitor UNI confirmed that GA can promote the bending of rhizome in *O. longistaminata*. That GA can overcome the sucrose effects suggest a cross-talk between sucrose and GA. Other report suggested that GA works as antagonist to sucrose, whereas abscisic acid (ABA) and jasmonate (JA) had a synergic effect with sucrose (Loreti et al. 2008). Cookson et al. (2016) reported that sugar depletion quickly induced the expression of photosynthesis and stress response genes while downregulating cell wall synthesis and signal transduction genes. This seems to be correlated with developmental transition from rhizomes to aerial stems as induced by the reduction in carbon, mainly sucrose. Further investigations of sucrose–hormone interaction and the repression mechanism of gene expression are needed to uncover the cross-linking association of such factors with sucrose, and to more fully understand the mechanisms regulating developmental transition in rhizomes.

## Conclusions

The developmental transition of rhizomes means the phenomenon occurring the identity shift from rhizome to aerial stem according with negative gravitropism. Our study shows that sucrose is the key factor that represses this phenomenon by downregulating the transcripts which are related to hormone and cell wall synthesis at the bending point in the *O. longistaminata* rhizomes.

An aerial stem, when laid on its side, starts an upward curvature within a day, whereas a rhizome maintains a horizontal elongation for 5 days before it gradually bends upward as if released from gravity. This suggests a gradual decrease/increase in whatever factor(s) is regulating negative gravitropism in the rhizome.

Rhizomes as a sink organ accumulate nutrients, particularly carbohydrates. Because the rhizome is incapable of photosynthesis, it relies on nutrient supply from the parental shoot to develop. We hypothesized that the point when the rhizome fails to obtain enough nutrient from the parent plant serves as a signal that activates developmental transition, so that a new ramet can shoot aboveground and produce energy via photosynthesis. Our examination showed that sucrose specifically repressed the developmental transition of rhizomes but the other sugars (glucose and fructose) did not. Moreover, expression analysis showed the effects of sucrose on the expression level of genes between the straight and bending part of the rhizome that are related to the developmental transition. These results indicate that sucrose is one of the important factors regulating the developmental transition of rhizomes in *O. longistaminata*.

## Electronic supplementary material

Below is the link to the electronic supplementary material.

**Supplementary material 1**. The rhizome bud penetrates the surrounding leaf sheath to elongate its internode horizontally. This movie was observed in hydroponic culture media by Brinno TLC200pro. (MOV 15795 KB)

**Supplementary material 2**. Elongating rhizome horizontally acquires negative gravitropism and goes upward to develop as a new individual. This movie was observed in hydroponic culture media by Canon EOS 40D. (MP4 8807 KB)

**Supplementary material 3**. Table S1, Figs. S1–S3 (PDF 4030 KB)

## References

[CR1] Azuma T, Inoue Y, Hamada Y, Okishio T, Sasayama D, Itoh K (2013). Anoxia promotes gravitropic curvature in rice pulvini but inhibits in wheat and oat pulvini. J Plant Physiol.

[CR2] Bel AJ, Hess PH (2008). Hexoses as phloem transport sugars: the end of a dogma?. J Exp Bot.

[CR3] Clore AM (2013). Cereal grass pulvini: agronomically significant models for studying gravitropism signaling and tissue polarity. Am J Bot.

[CR4] Coleman D, Fry B, Paul E, Melillo J (1991). Isotope ratios: I. sample preparation and mass spectrometric analysis. Carbon isotope techniques.

[CR5] Collings DA, Winter H, Wyatt SE, Allen NS (1998). Growth dynamics and cytoskeleton organization during stem maturation and gravity-induced stem bending in *Zea mays* L. Planta.

[CR6] Coneva V, Guevara D, Rothstein SJ, Colasanti J (2012). Transcript and metabolite signature of maize source leaves suggests a link between transitory starch to sucrose balance and the autonomous floral transition. J Exp Bot.

[CR7] Cookson SJ, Yadav UP, Klie S, Morcuende R, Usadel B, Lunn JE, Stitt M (2016). Temporal kinetics of the transcriptional response to carbon depletion and sucrose re-addition in *Arabidopsis* seedlings. Plant Cell Environ.

[CR8] Cosgrove DJ (1997). Cellular mechanisms underlying growth asymmetry during stem gravitropism. Planta.

[CR9] Dayanandan P, Kaufman PB (1984). Analysis and significance of gravity-induced asymmetric growth in the grass leaf-sheath pulvinus. Ann Bot.

[CR10] Drakakaki G, Zabotina O, Delgado I, Robert S, Keegstra K, Raikhel N (2006). *Arabidopsis* reversibly glycosylated polypeptides 1 and 2 are essential for pollen development. Plant Physiol.

[CR11] Evans MM, Poethig RS (1995). Gibberellins promote vegetative phase change and reproductive maturity in maize. Plant Physiol.

[CR12] Eveland AL, Jackson DP (2012). Sugars, signalling, and plant development. J Exp Bot.

[CR13] Fan Z, Cai Z, Shan J, Yang J (2017). Letter to the editor: bud position and carbohydrate play a more significant role than light condition in the developmental transition between rhizome buds and aerial shoot buds of *Oryza longistaminata*. Plant Cell Physiol.

[CR14] Figueroa CM, Lunn JE (2016). A tale of two sugars: trehalose 6-phosphate and sucrose. Plant Physiol.

[CR15] Gunning B, Sammut M (1990). Rearrangements of microtubules involved in establishing cell division planes start immediately after DNA synthesis and are completed just before mitosis. Plant Cell.

[CR16] Hu LW, Cui DY, Zang AP, Neill S, Cai WM (2009). Auxin-regulated OsRGP1 and OsSuS are involved in gravitropic bending of rice shoot bases. J Mol Cell Biol.

[CR17] Khush GS (1997). Origin, dispersal, cultivation and variation of rice. Plant Mol Biol.

[CR18] Koch K (2004). Sucrose metabolism: regulatory mechanisms and pivotal roles in sugar sensing and plant development. Curr Opin Plant Biol.

[CR19] Kumar D, Wareing PF (1972). Factors controlling stolon development in the potato plant. New Phytol.

[CR20] Lemoine R, La Camera S, Atanassova R, Dédaldéchamp F, Allario T, Pourtau N, Bonnemain JL, Laloi M, Coutos-Thévenot P, Maurousset L, Faucher M, Girousse C, Lemonnier P, Parrilla J, Durand M (2013). Source-to-sink transport of sugar and regulation by environmental factors. Front Plant Sci.

[CR21] Li P, Wang Y, Qian Q, Fu Z, Wang M, Zeng D, Li B, Wang X, Li J (2007). LAZY1 controls rice shoot gravitropism through regulating polar auxin transport. Cell Res.

[CR22] Long JC, Zhao W, Rashotte AM, Muday GK, Huber SC (2002). Gravity-stimulated changes in auxin and invertase gene expression in maize pulvinal cells. Plant Physiol.

[CR23] Loreti E, Povero G, Novi G, Solfanelli C, Alpi A, Perata P (2008). Gibberellins, jasmonate and abscisic acid modulate the sucrose-induced expression of anthocyanin biosynthetic genes in Arabidopsis. New Phytol.

[CR24] McDowell ET, Gang DR, Gang D (2013). A dynamic model for phytohormone control of rhizome growth and development. Phytochemicals, plant growth, and the environment.

[CR40] McIntyre GI (2000). Control of plant development by limiting factors: a nutritional perspective. Physiol Plant.

[CR25] Montaldi ER (1969). Gibberellin–sugar interaction regulating the growth habit of bermuda grass (*Cynodon dactylon* (L) Pers.). Cell Mol Life Sci.

[CR26] O’Neill DP, Ross JJ (2002). Auxin regulation of the gibberellin pathway in pea. Plant Physiol.

[CR27] Persia D, Cai G, Del Casino C, Faleri C, Willemse MT, Cresti M (2008). Sucrose synthase is associated with the cell wall of tobacco pollen tubes. Plant Physiol.

[CR28] Pfeiffer I, Kutschera U (1995). Sucrose metabolism and cell elongation in developing sunflower hypocotyls. J Exp Bot.

[CR29] Philosoph-Hadas S, Friedman H, Meir S (2005). Gravitropic bending and plant hormones. Vitam Horm.

[CR30] Rakusová H, Gallego-Bartolomé J, Vanstraelen M, Robert HS, Alabadí D, Blázquez MA, Benková E, Friml J (2011). Polarization of PIN3-dependent auxin transport for hypocotyl gravitropic response in *Arabidopsis thaliana*. Plant J.

[CR31] Rood SB, Kaufman PB, Abe H, Pharis RP (1987). Gibberellins and gravitropism in maize shoots: endogenous gibberellin-like substances and movement and metabolism of [3H] Gibberellin A20. Plant Physiol.

[CR32] Ross JJ, Wolbang CM (2008). Auxin, gibberellins, and the gravitropic response of grass leaf sheath pulvini. Plant Signal Behav.

[CR33] Sack FD (1997). Plastids and gravitropic sensing. Planta.

[CR34] Song I, Lu CR, Brock TG, Kaufman PB (1988). Do starch amyloplasts act as the gravisensors in cereal grass pulvini?. Plant Physiol.

[CR35] Song K, Yeom E, Lee SJ (2014). Real-time imaging of pulvinus bending in *Mimosa pudica*. Sci Rep.

[CR36] Wolbang CM, Chandler PM, Smith JJ, Ross JJ (2004). Auxin from the developing inflorescence is required for the biosynthesis of active gibberellins in barley stems. Plant Physiol.

[CR37] Wong AY, Colasanti J (2007). Maize floral regulator protein INDETERMINATE1 is localized to developing leaves and is not altered by light or the sink/source transition. J Exp Bot.

[CR38] Wu LL, Song I, Kim D, Kaufman PB (1993). Molecular basis of the increase in invertase activity elicited by gravistimulation of oat-shoot pulvini. J Plant Physiol.

[CR39] Yoshida A, Terada Y, Toriba T, Kose K, Ashikari M, Kyozuka J (2016). Analysis of rhizome development in *Oryza longistaminata*, a wild rice species. Plant Cell Physiol.

