# Correction to: Sucrose affects the developmental transition of rhizomes in *Oryza longistaminata*

**DOI:** 10.1007/s10265-019-01112-y

**Published:** 2019-04-29

**Authors:** Kanako Bessho‑Uehara, Jovano Erris Nugroho, Hirono Kondo, Rosalyn B. Angeles-Shim, Motoyuki Ashikari

**Affiliations:** 1 Bioscience and Biotechnology Center, Nagoya University, Furo-cho, Chikusa, Nagoya, Aichi 464-8601 Japan; 2 Department of Plant and Soil Science, Texas Tech University, Lubbock, TX 79409 USA

## Correction to: Journal of Plant Research (2018) 131:693–707 10.1007/s10265-018-1033-x

The article Sucrose affects the developmental transition of rhizomes in *Oryza longistaminata*, written by Kanako Bessho-Uehara, Jovano Erris Nugroho, Hirono Kondo, Rosalyn B. Angeles-Shim, Motoyuki Ashikari, was originally published electronically on the publisher’s internet portal (currently SpringerLink) on 8 May 2018 without open access.

With the author(s)’ decision to opt for Open Choice the copyright of the article changed on 5 May 2019 to © The Author(s) 2019 and the article is forthwith distributed under the terms of the Creative Commons Attribution 4.0 International License (http://creativecommons.org/licenses/by/4.0/), which permits use, duplication, adaptation, distribution and reproduction in any medium or format, as long as you give appropriate credit to the original author(s) and the source, provide a link to the Creative Commons license and indicate if changes were made.

Open Access This article is distributed under the terms of the Creative Commons Attribution 4.0 International License (http://creativecommons.org/licenses/by/4.0/), which permits unrestricted use, distribution, and reproduction in any medium, provided you give appropriate credit to the original author(s) and the source, provide a link to the Creative Commons license, and indicate if changes were made.

The original article was corrected.

