# Supplementary material for: Sucrose affects the developmental transition of rhizomes in Oryza longistaminata
Source: J Plant Res. 2018 May 8;131(4):693–707. doi: 10.1007/s10265-018-1033-x (PMC6488557; doi:10.1007/s10265-018-1033-x)
Supplement: Supplementary file 3 — Supplementary material 3. Table S1, Figs. S1–S3 (PDF 4030 KB) [file 10265_2018_1033_MOESM3_ESM.pdf]

Electric supplementary materials

Title:

Sucrose affects the developmental transition of rhizomes in *Oryza longistaminata*

Authors:

Kanako Bessho-Uehara[1†], Jovano Erris Nugroho[1†], Hirono Kondo[1], Rosalyn B. Angeles-Shim[1,2] and Motoyuki Ashikari[1*]

Journal:

Journal of Plant Research

Corresponding author:

Motoyuki Ashikari (Bioscience and Biotechnology Center, Furo-cho, Chikusa, Nagoya, Aichi 464-8601, Japan)

Tel: +81-52-789-5202

Fax: +81-52-789-5206

e-mail: ashi@agr.nagoya-u.ac.jp

Content:

Table S1

Figs. S1–S3

Table S1. Primers used in this paper

Purpose	Name	Sequence
qRT-PCR	OsSUT1_F	ATCTCCATCGTCGTCCCTCA
	OsSUT1_R	TTGGAGATCTTGGGCAGCAG
	GA20ox2_F	CGCCGCCTTCCAATTTT
	GA20ox2_R	AAGCCCAACCCAACCCA
	GA2ox3_F	ATGGATGAACAGCCTCCAATAGTT
	GA2ox3_R	CGAGAAAATCAAAGGCAAGAGG
	PIN1_F	AGCAAGCGTCCAGCCTTCAG
	PIN1_R	CTCTCCCCCACGCCGAACAG
	OsRGP1_F	GGGAGAGATTACTTAAGCAAC
	OsRGP1_R	CATTTAGCAACCAAAGCAC
	OsSUS1_F	GGCTGAGACTCTGAAAGAGG
	OsSUS1_R	CCTAGGAATGCAAAGCATC
	Ivr2 _F	GTCGGCAAAGCAACACAG
	Ivr2 _R	CGCAGCTGTATGCTGCTG

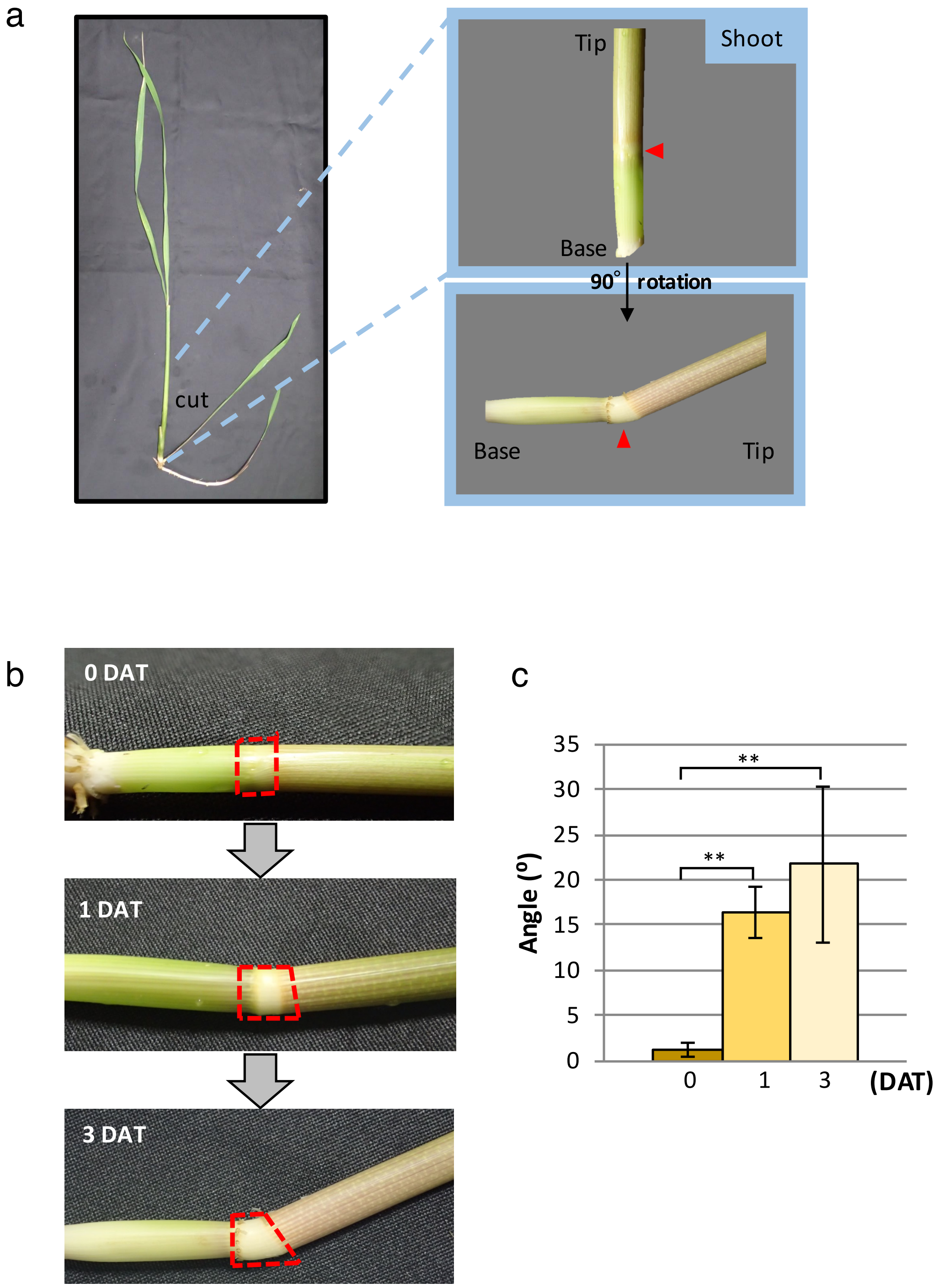

Fig. S1 Gravitropic stimulation of *O. longistaminata* aerial stem. (a) Illustration of how *O. longistaminata* aerial stem was excised and orientated to lie onto its side. Red triangle indicates pulvinus on aerial stem. (b) Representative pictures of stem bending; red dotted lines highlight pulvinus. DAT: days after treatment (c) The change of angle in aerial stem by negative gravitropism. Graph shows the mean of n=6 plants and error bars indicate ±SD. Significant difference were detected by student t-test and $**p \leq 0.01$.

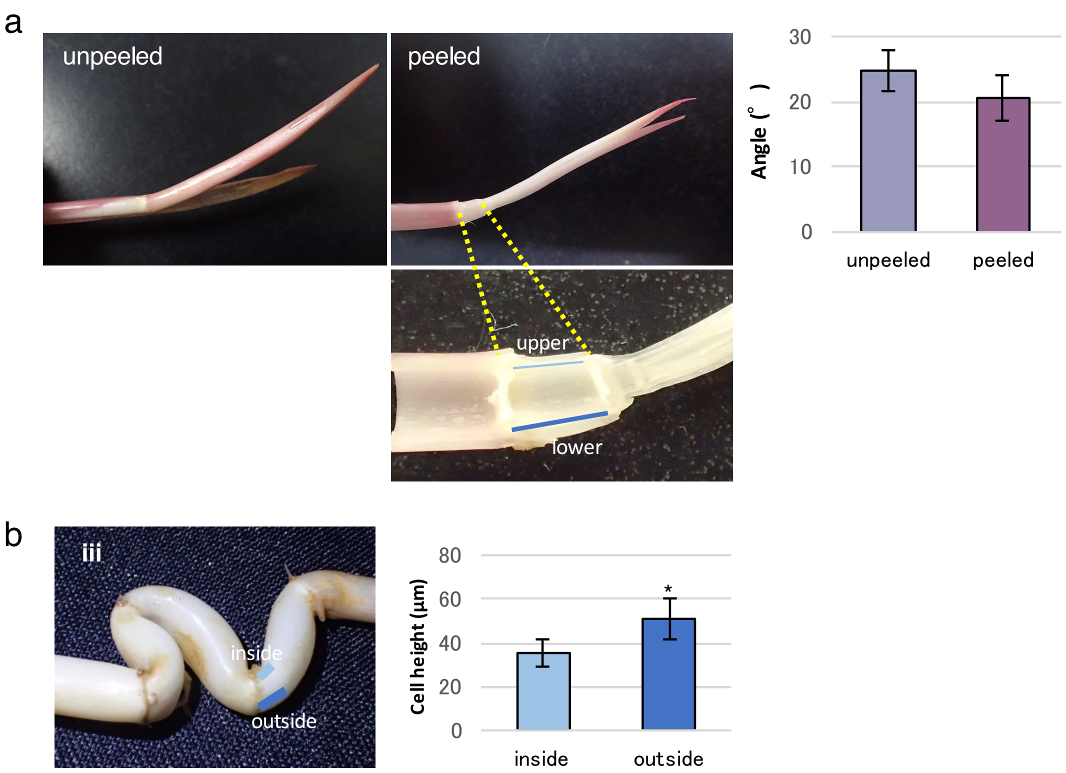

Fig. S2 Rhizome bending initiated at the internode. (a) The rhizome tip pictures unpeeled or peeled of scale leaves around 6th node from the base. Magnified picture shows the longitudinal section of the internode, with the upper part of the internode evidently shorter than the lower part. The graph shows the bending angle at this point from 5 samples. (b) A similar picture is depicted in Fig. 2b (iii). A graph shows the cell height from 20 cells of 3 samples. Error bars indicate ±SD. Significant difference was detected by student t-test, * $p \leq 0.05$.

a

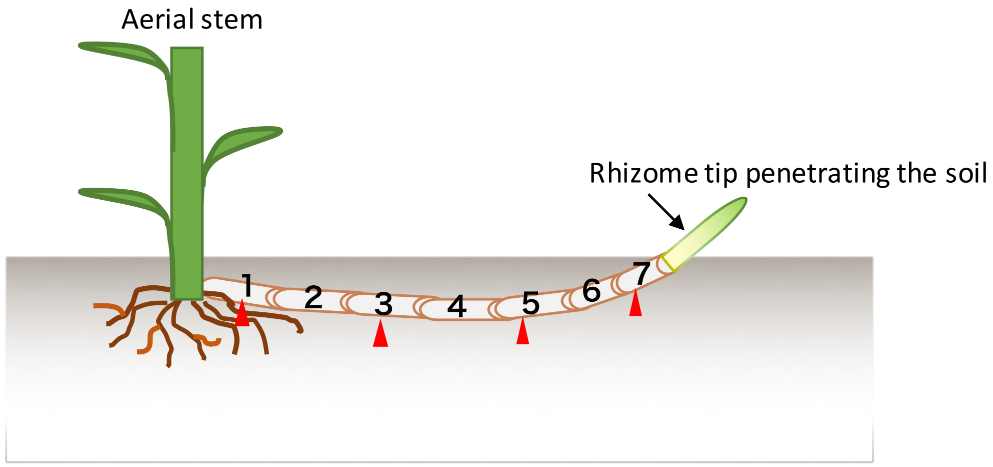

b

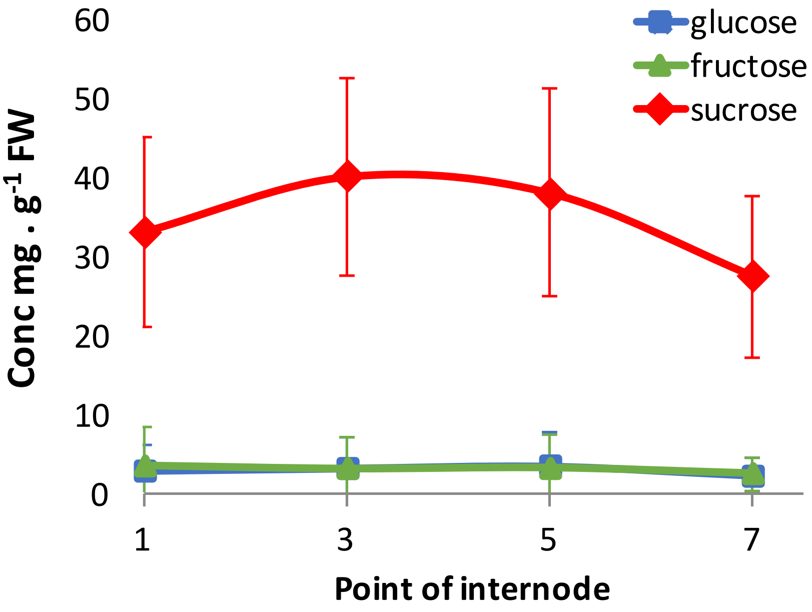

Fig. S3 Amount of sugar in *O. longistaminata* rhizome that penetrates the soil surface. (a) Illustration of rhizome sampling points. The numbers pointed by red triangle indicate sampling internodes (1st, 3rd, 5th, 7th) in the rhizome. (b) Concentration of sucrose, glucose and fructose along the rhizomes. Error bars indicate ±SD for 5 biological replicates.
